# Supplementary material for: Visualizing the enzyme mechanism of mevalonate diphosphate decarboxylase
Source: Nat Commun. 2020 Aug 7;11:3969. doi: 10.1038/s41467-020-17733-0 (PMC7414129; doi:10.1038/s41467-020-17733-0)
Supplement: Supplementary file 1 — Supplementary Information [file 41467_2020_17733_MOESM1_ESM.pdf]

Supplementary Information

Visualizing the Enzyme Mechanism of Mevalonate Diphosphate Decarboxylase

Chun-Liang Chen et al.

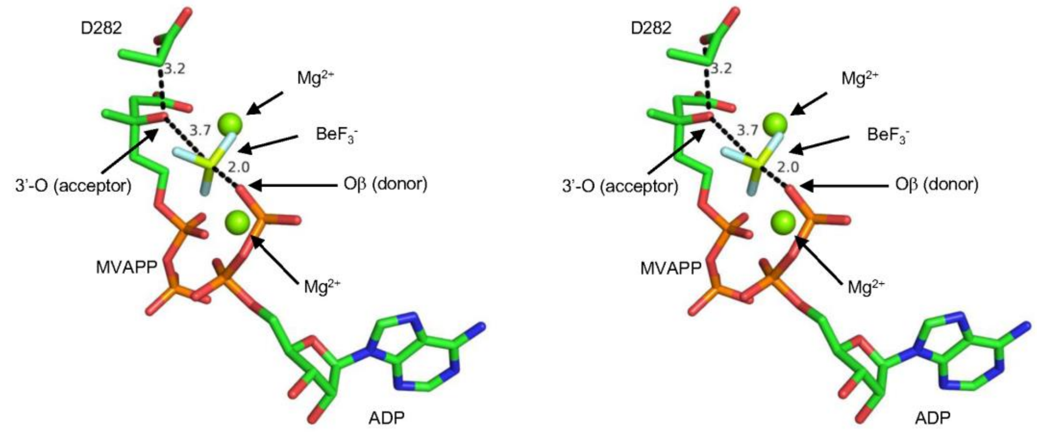

Supplementary Figure 1 The stereo view of the ligands (MVAPP, ADPBeF$_3$ and Mg^{2+}) in the active site of MDD$_{EF}$-MVAPP-ADPBeF$_3$-Mg^{2+} (PDB: 6E2V). The distance between the in-line phosphoryl transfer donor (Oβ of ADP) and beryllium of BeF$_3$ is 2.0 Å. The distance between the in-line phosphoryl transfer acceptor (3’-O of MVAPP) and beryllium of BeF$_3$ is 3.7 Å. The distance between the sidechain carboxyl group of D282 and 3’-O of MVAPP is 3.2 Å.

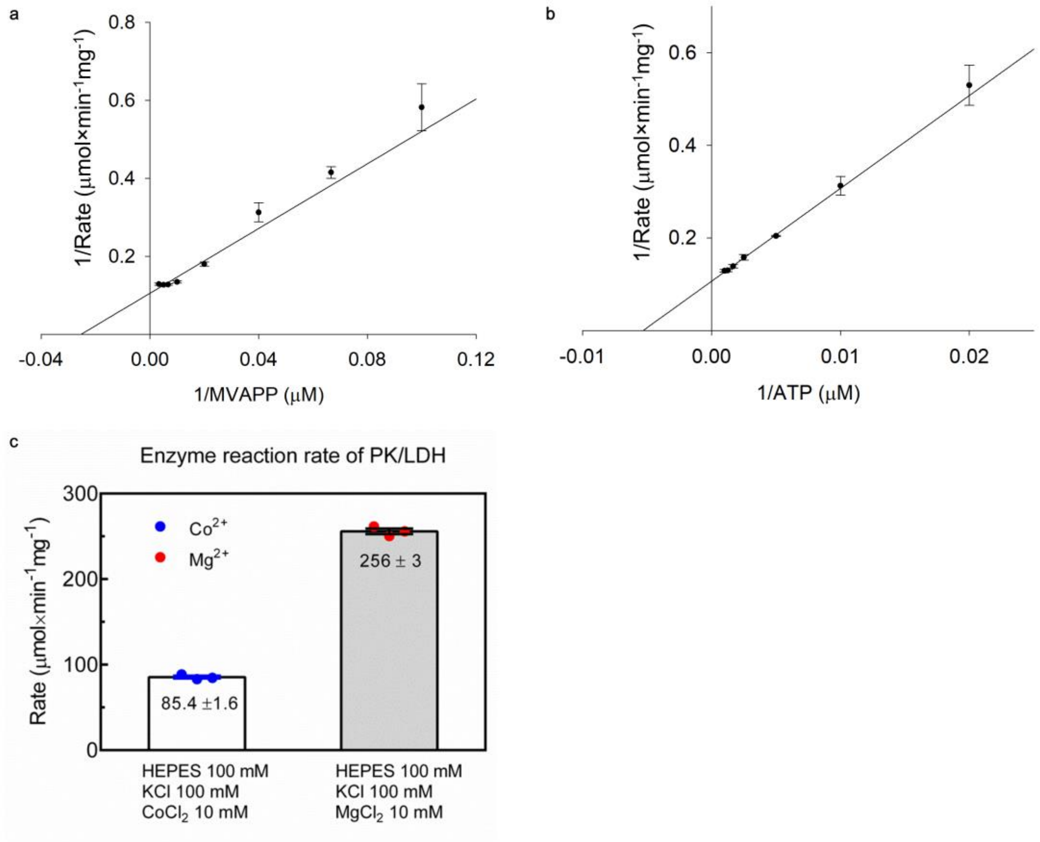

Supplementary Figure 2 Kinetic study of MDD_{EF} with two substrates under conditions with Co^{2+}. (a) Enzymatic reactions (n=3 independent experiments) were performed at varying concentrations of MgATP (50, 100, 200, 400, 600, 800 and 1000 μM) and a saturating concentration of MVAPP (300 μM). Kinetics data were fitted into the Henri & Michaelis & Menten equation (Supplementary Note 1, Supplementary Equation 3). K_{mMgATP} is 188 ± 13 μM and V_{max} is 9.5 ± 0.3 μmol×min^{-1}mg^{-1}. Source data are provided as a Source Data file. **(b)** Enzymatic reactions (n=3 independent experiments) were performed at varying concentrations of MVAPP (10, 15, 25, 50, 100, 200 and 300 μM) and a saturating concentration of MgATP (1000 μM) and the K_{mMVAPP} value is 39.3 ± 4.0 μM. Source data are provided as a Source Data file. **(c)** The values of capacities of enzyme-coupled reactions with the addition of either cobalt (10 mM) or magnesium (10 mM) were determined at fixed concentrations of ADP (400 μM), phosphoenolpyruvate (400 μM) and NADH (400 μM) (n=3 independent experiments). The assay under the cobalt conditions shows a rate of enzymatic reaction up to 85.4 ± 1.6 μmol×min^{-1}mg^{-1}, and the assay under the magnesium conditions a rate of enzymatic reaction up to 256 ± 3 μmol×min^{-1}mg^{-1}. Source data are provided as a Source Data file. The kinetic parameters are listed in Supplementary Table 1. Error bars represent standard error of the mean (SEM).

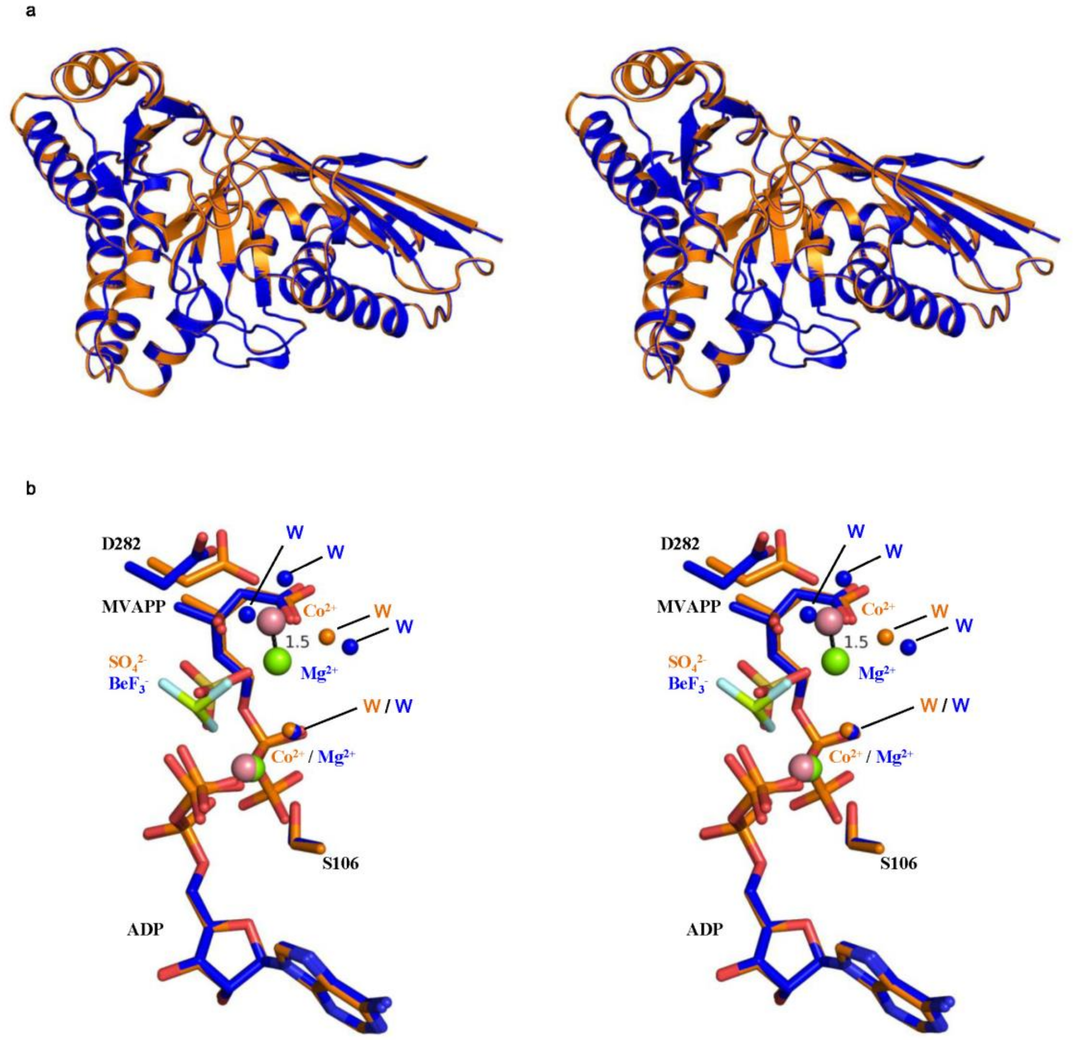

Supplementary Figure 3 The structural comparison of MDD_{EF}-MVAPP-ADP-SO_4^{2-}-Co^{2+} (PDB: 6E2W) and MDD_{EF}-MVAPP-ADPBeF_3-Mg^{2+} (PDB: 6E2V). (a) The stereo view of the structural superposition of MDD_{EF}-MVAPP-ADP-SO_4^{2-}-Co^{2+} (orange) and MDD_{EF}-MVAPP-ADPBeF_3-Mg^{2+} (blue). **(b)** The superimposition of ligands of two structures is shown in stereo with residues and molecules labeled. "W" represents ordered water molecules in the active site of the two structures.

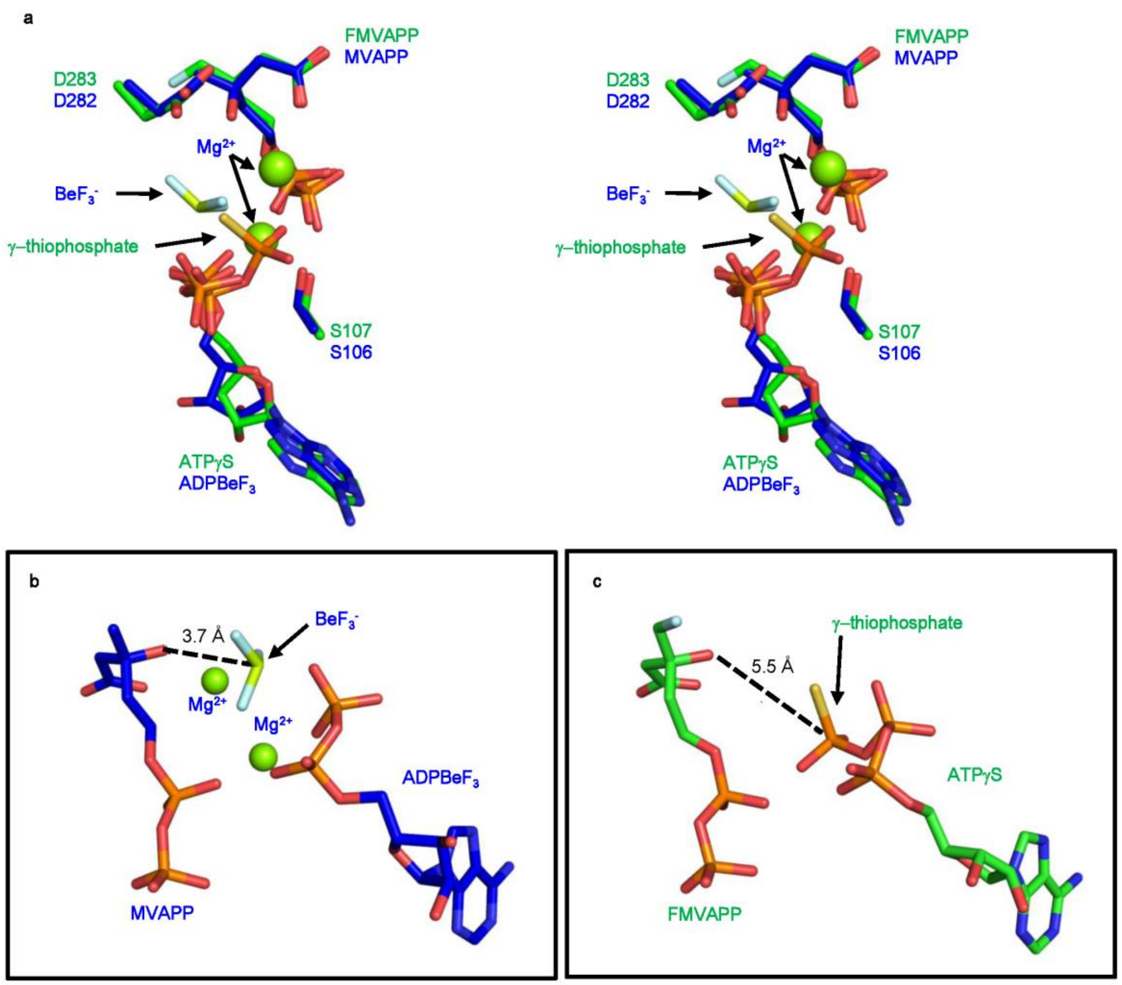

Supplementary Figure 4 The structural models of MDD from *Staphylococcus epidermidis* (MDD$_{SE}$-FMVAPP-ATPγS, PDB: 4DPT) and *Enterococcus faecalis* (MDD$_{EF}$-MVAPP-ADPBeF$_3$-Mg^{2+}, PDB: 6E2V). (a) The stereo view of the superposition of MDD$_{SE}$-FMVAPP-ATPγS and MDD$_{EF}$-MVAPP-ADPBeF$_3$-Mg^{2+}. The conserved aspartate and serine residues in both structures (D283 and S107 in MDD$_{SE}$-FMVAPP-ATPγS; D282 and S106 in MDD$_{EF}$-MVAPP-ADPBeF$_3$-Mg^{2+}) are indicated and shown as stick models. **(b)** Ligands of MDD$_{EF}$-MVAPP-ADPBeF$_3$-Mg^{2+} (MVAPP, ADP, BeF$_3^-$ and 2 magnesium ions (Mg^{2+}) in the active site. The distance between the 3'-oxygen of MVAPP and the γ-phosphorus of ATPγS is 3.7 Å. **(c)** The ligands, FMVAPP and ATPγS, in the active site of MDD$_{SE}$-FMVAPP-ATPγS. The distance between the 3'-oxygen of MVAPP and the γ-phosphorus of ATPγS is 5.5 Å.

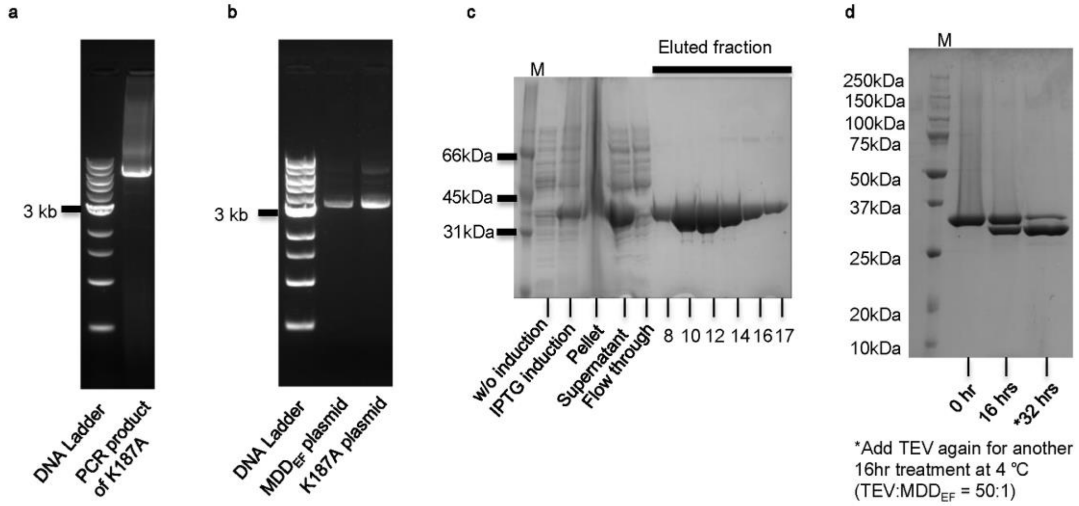

Supplementary Figure 5 Preparation of TEV-protease-treated MDD$_{EF}$ and the K187A mutant proteins. (a) The K187A mutant was created by site-directed mutagenesis and the PCR product of the K187A mutant was analyzed by agarose gel electrophoresis (lane 2). **(b)** The extracted plasmid of the K198A mutant after *E. coli* transformation. The DNA ladder was loaded in the first lane and the 3-kb position is indicated. The plasmid of MDD$_{EF}$ was loaded in the second lane for estimating the size of the PCR product of the K187A-mutant plasmid. **(c)** SDS-PAGE analysis for the K187A mutant protein purification. (Cell pellets before induction (w/o induction), after IPTG induction (IPTG induction), pellet (Pellet) and supernatant (supernatant) after French press, protein solution after the Ni^{2+} column (flow through) and protein eluted fractions (8, 10, 12, 14, 16 and 17) were examined by SDS-PAGE after protein purification). **(d)** SDS-PAGE analysis of the TEV-protease-treated K187A mutant protein. **(a)**, **(b)**, **(c)** and **(d)** were replicated only once.

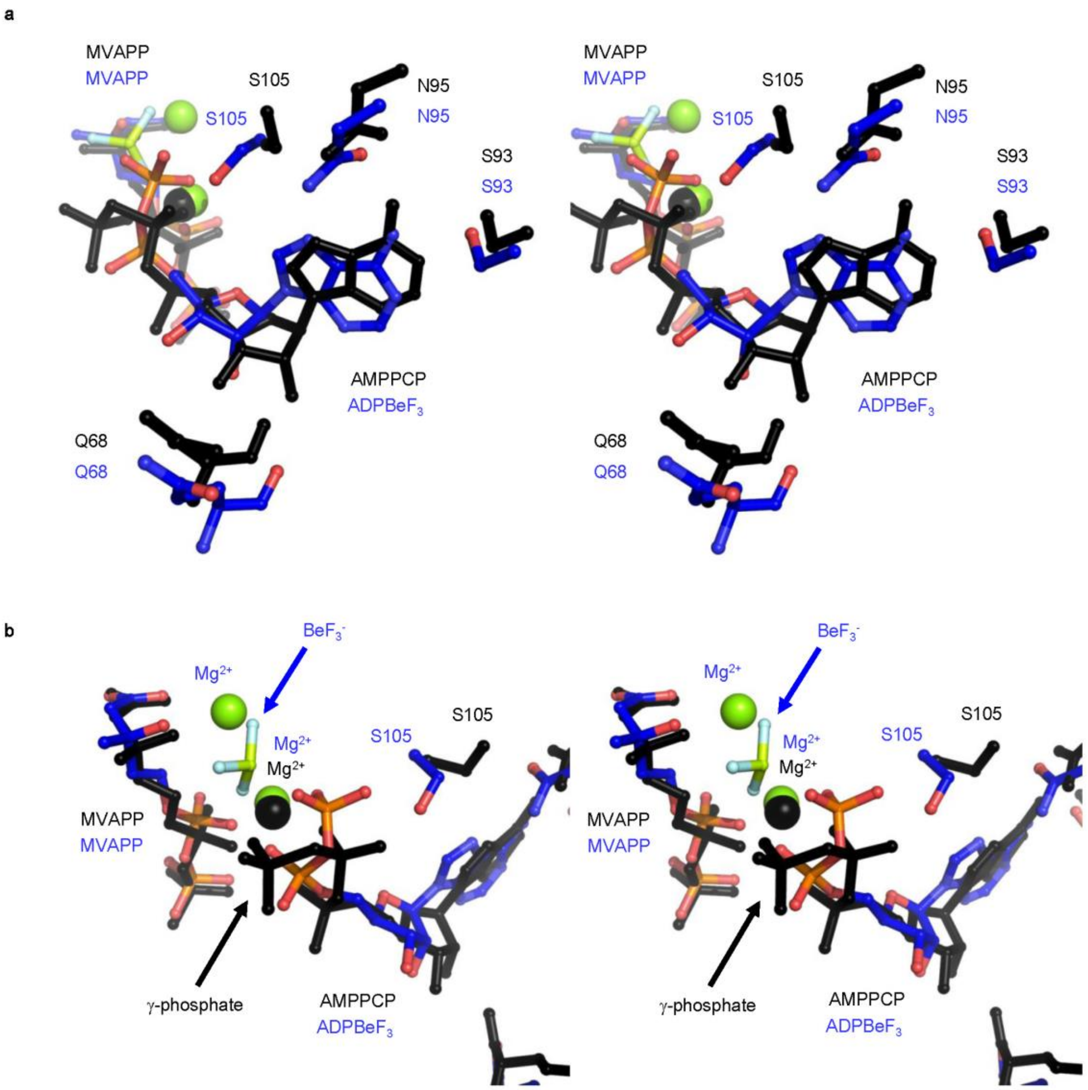

Supplementary Figure 6 Structural comparison of ligands in the active site of MDD$_{EF}$-MVAPP-AMPPCP-Mg^{2+} (PDB: 6E2U) and MDD$_{EF}$-MVAPP-ADPBeF$_3$- Mg^{2+} (PDB: 6E2V). Ligands and residues involved in the binding of ATP from MDD$_{EF}$-MVAPP-AMPPCP-Mg^{2+} are colored in black. Ligands and residues from MDD$_{EF}$-MVAPP-ADPBeF$_3$-Mg^{2+} are colored in blue. **(a)** The figure is positioned to emphasize the differences between the adenosine moieties of AMPPCP and ADPBeF$_3$ in these two structures. **(b)** The figure is positioned to emphasize the differences between the γ-phosphate of AMPPCP and BeF$_3^-$ (the γ-phosphate mimic) of ADPBeF$_3$ in these two structures.

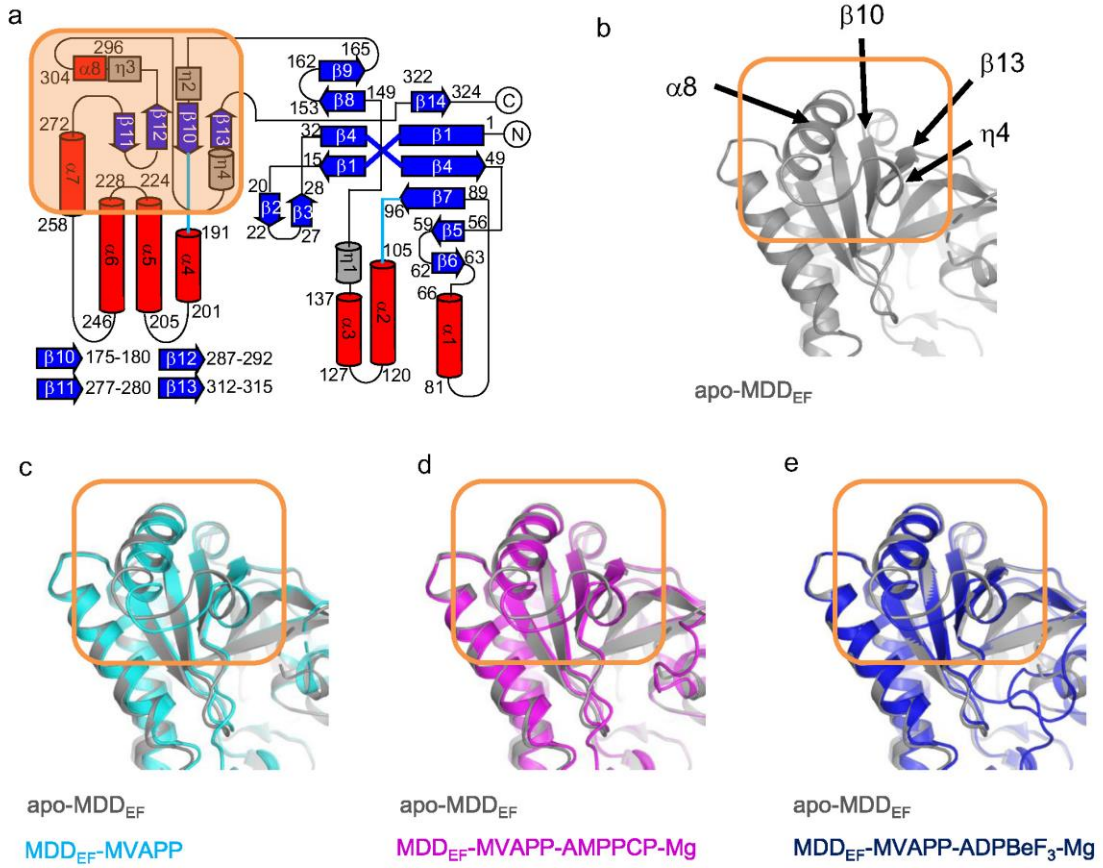

Supplementary Figure 7 Secondary structure elements around the β10 strand of MDD$_{EF}$. (a) Topology of MDD$_{EF}$. The range of amino acid numbers of each secondary structural element is described in Fig. 1. The area shown in orange contains the α8, η4, β13 elements of MDD$_{EF}$ which move down upon MVAPP binding and the presence of the β10-α4 loop. **(b)** An local area of apo-MDD$_{EF}$ with labeled α8, η4, β10 and β13 secondary structure elements. (c), (d) and (e) show the structural comparison between the apo-MDD$_{EF}$ and MDD$_{EF}$ ligand-bound structures in the α8-η4-β13 area (orange box). The followings list the MDD$_{EF}$ structures and PDB accession codes in parentheses: apo-MDD$_{EF}$ (6E2S); MDD$_{EF}$-MVAPP (6E2T); MDD$_{EF}$-MVAPP-AMPPCP-Mg (6E2U); MDD$_{EF}$-MVAPP-ADPBeF$_3$-Mg (6E2V).

Supplementary Table 1 Comparison of published MDD structures

Species in bacteria	Ligand	β-α loop*	α8 η4 β13/full length α4**	P-loop[#]	PDB	Cryo-protectant	Space group	Direct crystal packing/contact
MDD *E. faecalis*[1]	---	---	up/no	---	5V2M	$(NH_4)_2SO_4$/Glycerol (pH 4.6)	$P2_12_12$	
MDD *E. faecalis*[1]	ATP	---	up/no	open	5V2L	PEG400/3350 (pH 4.6)	$P2_12_12$	
MDD *E. faecalis*	---	---	up/no	---	6E2S	PEG400/3350 (pH 4.6)	$P2_12_12$	
MDD *E. faecalis*	MVAPP	open	down/yes	---	6E2T	PEG400/3350 (pH 4.6)	$P2_12_12$	
MDD *E. faecalis*	MVAPP, AMPPCP, Mg^{2+}	open	down/yes	open	6E2U	PEG400/3350 (pH 4.6)	$P2_12_12$	
MDD *E. faecalis*	MVAPP, ADP, BeF_3, Mg	closed	down/yes	closed	6E2V	PEG400/3350 (pH 4.6)	$P2_12_12$	
MDD *E. faecalis*	MVAPP, ADP, SO_4^{2-}, Co^{2+}	closed	down/yes	closed	6E2W	PEG400/3350 (pH 4.6)	$P2_12_12$	
MDD *S. pyogenes*	---	open	down/yes	open	2GS8	PEG3350 (pH 4.5)	$P2_12_12_1$	β-α loop, P-loop
MDD *S. epidermidis*[2]	---	---	down/no	open	3QT5	PEG3350 (pH 7.0)	$C222_1$	P-loop
MDD *S. epidermidis*[3]	MVAPP	open (has density but no model built in	down/yes	open	4DU7	PEG3350 (pH 7.0)	$C222_1$	β-α loop, P-loop
MDD *S. epidermidis*[2]	FMVAPP	open (has density but no model built in	down/yes	open	3QT7	PEG3350 (pH7.0)	$C222_1$	β-α loop, P-loop
MDD *S. epidermidis*[2]	DPGP	open	down/yes	open	3QT6	PEG3350 (pH 7.0)	$C222_1$	β-α loop, P-loop

MDD *S. epidermidis*[3]	FMVAPP, ATPγS	---	down/yes	close	4DPT	PEG3350 (pH 7.0)	*C*121	
MDD *S. epidermidis* D283A[3]	DPGP	open	down/yes	open	4DU8	PEG3350 (pH 7.0)	$C222_1$	β-α loop, P-loop
MDD *S. epidermidis* D283A[3]	MVAPP, ATPγS	closed	down/yes	closed	4DPW	PEG3350 (pH 7.0)	$P2_12_12_1$	
MDD *S. epidermidis* S192A[3]	---	open	down/yes	open	4DPX	PEG3350 (pH 7.0)	$C222_1$	β-α loop, P-loop
MDD *S. epidermidis* S192A[2]	FMVAPP	open (has density but no model built in	down/yes	open	3QT8	PEG3350 (pH 7.0)	$C222_1$	β-α loop, P-loop
MDD *S. epidermidis* S192A[3]	DPGP	open	down/yes	open	4DPY	PEG3350 (pH 7.0)	$C222_1$	β-α loop, P-loop
MDD *S. epidermidis* S192A[3]	FMVAPP ATPγS	open	down/yes	open	4DPU	PEG3350 (pH 7.0)	$C222_1$	β-α loop, P-loop
MDD *S. aureus*[4]	---	open	down/yes	open	2HK2	Malonate (pH 8.0)	$P12_11$	N-Tag interacts with both loops
MDD *S. aureus*[4]	---	open	down/yes	open	2HK3	Malonate (pH 8.0)	$P2_12_12_1$	N-Tag interacts with both loops
MDD *L. pneumophila*	---	open	down/yes	open	3LTO	PEGMME2000 (pH 4.6)	$P2_12_12_1$	P-loop

Species in eukaryotes	Ligand	β-α loop*	α8 η4 β13/full length α4**	P-loop#	PDB	Cryo-protectant	Space group	Direct crystal packing/contact
MDD *H. sapiens*[5]	---	open	down/yes	---	3D4J	$(NH_4)_2SO_4$ (pH 6.5)	$P12_11$	β-α loop
MDD *Mus musculus*	---	open	down/yes	open	3F0N	PEG3350 (pH 6.0)	$P4_32_12$	P-loop
MDD *A. thaliana*[6]	MVAPP	open	down/yes	open	6N10	$(NH_4)_2SO_4$ (pH 5.6)	$P4_12_12$	additional contacts with the C-terminal residues

MDD *S. cerevisiae*[7]	---	open	down/yes	open	1FI4	PEG4000 (pH 8.5)	$P2_12_12$	additional contacts with the C-terminal residues
MDD *T. brucei*[4]	---	open	down/yes	open	2HKE	PEG8000 (pH 6.0)	$P12_11$	P-loop
Species in archeae	Ligand	β-α loop*	α8 η4 β13/full length α4**	P-loop[#]	PDB	Cryo-protectant	Space group	Direct crystal packing/contact
MDD *S. solfataricus*[8]	---	half closed	down/yes	half closed (discontinuous density)	4Z7C	Glycerol (pH 7.5)	$H3_2$	
MDD *S. solfataricus*[8]	---	half closed	down/yes	half closed	4Z7Y	$(NH_4)_2SO_4$ (pH 4.6)	$P12_11$	P-loop
MDD *S. solfataricus*[9]	MVAPP, ATPγS	half closed	down/yes	half closed	5GMD	$(NH_4)_2SO_4$ (pH 4.6)	$H3_2$	P-loop
MDD *S. solfataricus*[9]	MVAPP, ADP	half closed	down/yes	half closed	5GME	$(NH_4)_2SO_4$ (pH 4.6)	$H3_2$	P-loop

*The β−α loop may be numbered differently in MDD proteins. In MDD_{EF}, it is the β10-α4 loop.
**α8 η4 β13 is named based on the secondary structure element in MDD_{EF}. Apo MDD_{EF} has these three elements in an "Up" position (Supplementary Figure 7b). All of other structures were superimposed on apo MDD_{EF}. The "down" position of these three elements means that these are closer to the active site and similar to other MDD_{EF}-ligand bound structures (Supplementary Figure 7c, 7d, 7e).
P-loop here stands for the phosphate binding loop.

Supplementary Table 2 Enzyme kinetic parameters*

V_{max} (μmol min^{-1}mg^{-1})	V_{max} (μMs^{-1})	k_{cat} (s^{-1})	K_{mMVAPP} (μM)	K_{mATP} (μM)	Metal
16.1 ± 0.3	0.75 ± 0.02	9.8 ± 0.2	39.7 ± 2.8	166 ± 12	Mg^{2+}
9.5 ± 0.3	0.44 ± 0.01	5.7 ± 0.2	39.3 ± 4.0	188 ± 13	Co^{2+}

* The means and the standard deviation of each data point were obtained from a triplicate test (Means ± the standard error). All the experiments were conducted at 30°C.

Supplementary Table 3 The interaction profile between MDD_{EF} and ligands in the ligand-bound MDD_{EF} structures*

Hydrogen bonds between ligands and MDD_{EF}				Crystal structure		
Atom of Ligand		Atom of MDD_{EF}		MDD_{EF}-MVAPP	MDD_{EF}-MVAPP-AMPPCP-Mg^{2+}	MDD_{EF}-MVAPP-$ADPBeF_3$-Mg^{2+}
MVAPP	O2, O3B	Y17	N, OH	2.9, 2.6	2.9, 2.5	2.9, 2.7
	O1B	K20	NZ	2.7	2.6	2.7
	O2B	K71	NZ	2.8	2.8	
	O2B	S138	OG	2.9	2.8	2.6
	O1B	G139	N	2.8	2.8	2.8
	O1A	S140	N, OG	3.0, 2.7	3.0, 2.6	2.9, 2.7
	O2, O1	R143	NH1, NH2	2.9, 2.9	3.0, 2.8	3.0, 3.0
	O2A	S191	OG	2.7	2.8	
	O2B, O1B	R192	NH2, NE	3.0, 2.7	3.0 , 2.8	2.8, 2.8
ATP, AMPPCP $ADPBeF_3$	O2'	Q68	O		2.8	
	O3'	Q68	O		2.7	
	O1A	K71	NZ		2.6	
	N6	S93	OG		3.3	3.0
	N6	N95	OD1		2.4	3.1
	N7	N95	ND2			3.1
	O3B	A100	N			3.1
	O2B	G102	N			3.1
	O2B	A104	N			3.4
	O3B	S105	N		3.2	3.0
	O3B	S105	OG			2.5
	O1A	S105	N			
	O1B	S106	N		3.2	2.9
	O2B (Bridging)	K187	NZ			3.0
	O1A	S191	N			3.0
	BEF(F2)	A104	N			2.9
	F3 or O2G	S191	OG		2.7	2.7
	F1 or O3G	A283	N			3.0
	O3G	S191	N		3.0	
Bonds between ligands and MDD_{EF}						
Atom of Ligand		Atom of MDD_{EF}				
Mg	MG	S106	OG		2.6	2.2

***** The MDD_{EF} structures and PDB accession codes in parentheses are listed as followings: apo-MDD_{EF} (6E2S); MDD_{EF}-MVAPP (6E2T); MDD_{EF}-MVAPP-AMPPCP-Mg^{2+} (6E2U); MDD_{EF}-MVAPP-$ADPBeF_3$-Mg^{2+} (6E2V).

Supplementary Table 4 Comparison of helix angles* in the unbound and bound forms of MDD_{EF}[#]

Crystal structure		Angle between helical positions from A and B (°)		
A	B	α1	α2	α4
MDD_{EF}-SO_4^{2-}	MDD_{EF}-MVAPP	4.5	4.2	10.9
MDD_{EF}-MVAPP	MDD_{EF}-MVAPP-AMPPCP-Mg^{2+}	0.7	2.0	0.7
MDD_{EF}-MVAPP-AMPPCP-Mg^{2+}	MDD_{EF}-MVAPP-ADPBeF_3-Mg^{2+}	9.7	0.6	0.9
MDD_{EF}-SO_4^{2-}	MDD_{EF}-MVAPP-ADPBeF_3-Mg^{2+}	5.8	6.1	10.7

* The angles were obtained using the command "angle_between_helices" in the Pymol script "AngleBetweenHelices" created by Thomas Holder.
[#] The MDD_{EF} structures and PDB accession codes in parentheses are listed as followings: apo-MDD_{EF} (6E2S); MDD_{EF}-MVAPP (6E2T); MDD_{EF}-MVAPP-AMPPCP-Mg^{2+} (6E2U); MDD_{EF}-MVAPP-ADPBeF_3-Mg^{2+} (6E2V)

Supplementary Table 5 List of the center and direction* of Helix α1, α 2 and α4 in the unbound and bound forms of MDD_{EF}#

Crystal structure	Helix	Center	Direction
**MDD_{EF}-SO_4^{2-}**	α1	(103.93, 223.27, 6.91)	(-0.68, 0.14, -0.72)
	α2	(95.34, 215.12, 8.11)	(-0.78, 0.49, -0.38)
	α4	(120.23, 208.60, -0.25)	(0.40, -0.34, -0.85)
**MDD_{EF}-MVAPP**	α1	(104.23, 222.51, 6.75)	(-0.66, 0.21, -0.72)
	α2	(95.33, 214.83, 7.99)	(-0.75, 0.56, -0.35)
	α4	(118.08, 209.48, 0.76)	(0.56, -0.35, -0.75)
**MDD_{EF}-MVAPP-AMPPCP-Mg^{2+}**	α1	(104.17, 222.64, 6.81)	(-0.66, 0.20, -0.73)
	α2	(95.38, 214.93, 7.90)	(-0.76, 0.57, -0.32)
	α4	(118.17, 209.38, 0.76)	(0.56, -0.34, -0.75)
**MDD_{EF}-MVAPP-$ADPBeF_3$-Mg^{2+}**	α1	(104.15, 223.77, 6.54)	(-0.72, 0.05, -0.69)
	α2	(95.44, 214.90, 7.86)	(-0.76, 0.57, -0.31)
	α4	(117.98, 209.41, 1.14)	(0.56, -0.33, -0.76)

* The center and direction of the helices were obtained using the command "helix_orientation_hbond" in the Pymol script "AngleBetweenHelices" created by Thomas Holder.
# The MDD_{EF} structures and PDB accession codes in parentheses are listed as followings: apo-MDD_{EF} (6E2S); MDD_{EF}-MVAPP (6E2T); MDD_{EF}-MVAPP-AMPPCP-Mg^{2+} (6E2U); MDD_{EF}-MVAPP-$ADPBeF_3$-Mg^{2+} (6E2V).

Supplementary Table 6 Thermodynamic parameters of the K187A mutant

Sup. Table 1 Thermodynamic parameters*

	Species	Substrate	K_d (µM)	ΔG** (kcal/mol)	ΔH (kcal/mol)	TΔS# (kcal/mol)	ΔS (cal/mol*K)
a	K187A (100 µM)	MVAPP (1 or 2 mM)	8.7 ± 5.4	-7.1 ± 0.3	-0.6 ± 0.1	6.5 ± 0.2	21.9 ± 0.7
b	K187A (100 µM)	ATP (3 mM)	495 ± 91	-4.5 ± 0.1	-4.4 ± 1.5	0.1 ± 0.9	0.4 ± 2.9
c	K187A (100 µM)	ATPγS (3 mM)	182 ± 36	-5.1 ± 0.1	-6.3 ±0.4	-1.2 ± 0.2	-4.0 ± 0.8
d	K187A (100 µM) + MVAPP (1 mM)	ATPγS (2 mM)	58.2 ± 13.2	-5.8 ± 0.1	-2.1 ± 0.4	3.7 ± 0.2	12.4 ± 0.8

*Titration experiments were done at 25°C.

** The mean of ΔG (μ) is derived from the equation: $\mu = -\mathrm{RTln}\left(\frac{m}{\sqrt{1+\frac{v}{m^2}}}\right)$ and the standard deviation of ΔG (σ) is calculated from the equation: $\sigma = \sqrt{\ln\left(1+\frac{v}{m^2}\right)}$, where m is the mean of the association constant (K_a) and v is the variance of K_a derived from each ITC experiment.

The mean of TΔS (μ_{1-2}) is derived from the difference between the means of ΔG (μ_1) and ΔH (μ_2) and the standard deviation (σ_{1-2}) of TΔS is derived from the equation: $\sigma_{1-2} = \sqrt{\frac{\sigma_1{}^2}{n} + \frac{\sigma_2{}^2}{n}}$, where σ_1 is the standard deviation of ΔG and σ_2 is the standard deviation of ΔH.

Supplementary Note1

$$D_{(n)} = D_{(1)} - 0.6 \times \log(n)$$

Supplementary Equation 1

where $D_{(n)}$ is the half distance between the phosphoryl donor and acceptor, $D_{(1)}$ is the single bond distance (1.73 Å for the P-O bond) and n is the bonding order.

$$\mathrm{pH} = \mathrm{pKa} - \log\frac{[\mathrm{HA}]}{[\mathrm{A}^-]}$$

Supplementary Equation 2

Where pKa is the acid dissociation constant; [HA] is the protonated state of A; $[A^-]$ is the deprotonated state of A.

$$v = \frac{V_{\mathrm{max}}[\mathrm{S}]}{K_{\mathrm{m}} + [\mathrm{S}]}$$

Supplementary Equation 3

where *V*max is the maximum velocity; [S] is the substrate concentration; *K*m is the Michaelis-Menten constant.

Supplementary References

1. Chen, C.L., Mermoud, J.C., Paul, L.N., Steussy, C.N. & Stauffacher, C.V. Mevalonate 5-diphosphate mediates ATP binding to the mevalonate diphosphate decarboxylase from the bacterial pathogen. *J Biol Chem* **292**, 21340-21351 (2017).
2. Barta, M.L. et al. Crystal Structures of Staphylococcus epidermidis Mevalonate Diphosphate Decarboxylase Bound to Inhibitory Analogs Reveal New Insight into Substrate Binding and Catalysis. *Journal of Biological Chemistry* **286**, 23900-23910 (2011).
3. Barta, M.L., McWhorter, W.J., Miziorko, H.M. & Geisbrecht, B.V. Structural Basis for Nucleotide Binding and Reaction Catalysis in Mevalonate Diphosphate Decarboxylase. *Biochemistry* **51**, 5611-5621 (2012).
4. Byres, E., Alphey, M.S., Smith, T.K. & Hunter, W.N. Crystal structures of Trypanosoma brucei and Staphylococcus aureus mevalonate diphosphate decarboxylase inform on the determinants of specificity and reactivity. *J Mol Biol* **371**, 540-53 (2007).
5. Voynova, N.E. et al. Human mevalonate diphosphate decarboxylase: characterization, investigation of the mevalonate diphosphate binding site, and crystal structure. *Arch Biochem Biophys* **480**, 58-67 (2008).
6. Thomas, S.T., Louie, G.V., Lubin, J.W., Lundblad, V. & Noel, J.P. Substrate Specificity and Engineering of Mevalonate 5-Phosphate Decarboxylase. *ACS Chem Biol* **14**, 1767-1779 (2019).
7. Bonanno, J.B. et al. Structural genomics of enzymes involved in sterol/isoprenoid biosynthesis. *Proc Natl Acad Sci U S A* **98**, 12896-901 (2001).
8. Hattori, A. et al. In Vivo Formation of the Protein Disulfide Bond That Enhances the Thermostability of Diphosphomevalonate Decarboxylase, an Intracellular Enzyme from the Hyperthermophilic Archaeon Sulfolobus solfataricus. *J Bacteriol* **197**, 3463-71 (2015).
9. Motoyama, K. et al. A Single Amino Acid Mutation Converts (*R*)-5-Diphosphomevalonate Decarboxylase into a Kinase. *J Biol Chem* **292**, 2457-2469 (2017).
